# MicroRNA 210 Mediates VEGF Upregulation in Human Periodontal Ligament Stem Cells Cultured on 3DHydroxyapatite Ceramic Scaffold

**DOI:** 10.3390/ijms19123916

**Published:** 2018-12-06

**Authors:** Jacopo Pizzicannella, Marcos Cavalcanti, Oriana Trubiani, Francesca Diomede

**Affiliations:** 1 Department of Medical, Oral and Biotechnological Sciences, University “G. d’Annunzio” Chieti-Pescara, 66100 Chieti, Italy; jacopo.pizzicannella@unich.it (J.P.); francesca.diomede@unich.it (F.D.); 2 ASL02 Lanciano-Vasto-Chieti, Chieti “Ss. Annunziata” Hospital, 66100 Chieti, Italy; 3 Faculté de Médecine, UMR 7365 CNRS-Université de Lorraine, 9, Avenue de la Forêt de Haye, 54500 Vandoeuvre-lés-Nancy, France; mxistocavalcanti@gmail.com (M.C.); 4 Laser in Dentistry Program, Cruzeiro do Sul University (UNICSUL), 08060-070 Sao Paulo-SP, Brazil

**Keywords:** human periodontal ligament stem cells, bone substitutes, vascular endothelial growth factors, miR-210, osteogenesis

## Abstract

The aim of the present research was the evaluation of the behavior of human periodontal ligament stem cells (hPDLSCs), cultured in presence of Endobon^®^ Xenograft Granules (G), a fully deproteinated hydroxyapatite ceramic scaffold derived from cancellous bovine bone. hPDLSCs were seeded with and without G for 24 h to 1 week. The cell growth, morphological features, adhesiveness, differentiation ability, modulation of miR-210 and Vascular Endothelial Growth Factor (VEGF) secretion were analyzed by means of MTT assay, Scanning Electron Microscopy (SEM), Confocal Laser Scanning Microscopy (CLSM), Alizarin Red S assay, RT-PCR and ELISA test, respectively. hPDLSCs grown on the biomaterial showed the ability to form focal adhesion on the substrate, as demonstrated by vinculin expression. These data were supported by SEM analysis showing that an adhesiveness process associated to cell growth occurs between cells and biomaterials. The osteogenic differentiation, evaluated by morphological, biochemical, and RT-PCR analysis, was pronounced in the hPDLSCs grown in the three-dimensional inorganic bovine bone substitute in the presence of osteoinductive conditions. In addition, an upregulation of miR-210 and VEGF was evident in cells cultured in presence of the biomaterial. Our results inspire us to consider granules not only an adequate biocompatible three-dimensional biomaterial, but also an effective inductor of miR-210 and VEGF; in fact, the involvement of miR-210 in VEGF secretion could offer a novel regulatory system in the early steps of the bone-regeneration process.

## 1. Introduction

Periodontal disease is a wide range of inflammatory diseases leading to bone and tooth loss [1,2]. Nowadays, tissue engineering represents a novel approach to repair bone-tissue defects in oral, orthopedic, and maxillofacial surgery. In particular, tissue engineering in the bone-regeneration field shows significant limitations that affect current treatment options and clinical demand for bone grafts. Tissue engineering is based on two principal actors: the biomaterial and the kinds of cells.

The ideal scaffold is composed of a biocompatible, biodegradable material with similar mechanical features to the natural bone, it should also be with a porous structure and an interpored connection, and pose good mechanical strength to support load-bearing [3,4].

The essential components needed in bone-tissue engineering for successful results are represented by an appropriate scaffold and a suitable stem-cell source [5,6]. Human Mesenchymal Stem Cells (hMSCs) are a type of adult stem cell with multipotent characteristics that are easy to manipulate in vitro [7]. Mesenchymal stem cells are available and exist in a wide range of tissue, as bone marrow, cord blood, cartilage, tendons, and dental tissue. Bone-ligament periodontal complex regeneration showed unpredictable clinical outcomes and remains a challenge in dentistry [8]. We have recently reported that stem cells derived from human periodontal ligament (hPDLSCs) can be an easy and efficient autologous source of stem cells with high expansion capacity and the ability to differentiate in osteogenic cells that can colonize and grow connected to a biocompatible scaffold [9,10,11,12,13]. In addition, tissue regeneration needs an easy and efficient source of stem cells combined with an appropriate scaffold. Other important elements that have a key role in the bone-regeneration process are represented by growth and differentiation factors, which, by autocrine/paracrine mechanisms, can mediate reparative and regenerative actions. Among these proteins, Vascular Endothelial Growth Factor (VEGF) is known to act as promoter of angiogenesis [14], which plays a pivotal role in regenerative processes.

Indeed, VEGF has been shown to trigger the development of new blood vessels from pre-existing capillaries and to enhance their permeability [15,16], thus triggering the first steps in tissue regeneration by favoring the supply of oxygen and nutrients and by facilitating the migration of cells into the engineered bone [17]. Furthermore, VEGF is directly involved in several aspects of bone development and remodeling by promoting the differentiation and recruitment of osteoblasts and osteoclasts [18]. The recent literature showed an important role of miR-210 for cell survival and angiogenesis.

MicroRNAs (miRNAs) play a role in the regulation of protein levels involved in the transduction of angiogenic signals. miRNAs are short noncoding RNAs, composed of 20–22 nucleotides, which regulate gene expression in a variety of physiological and pathological conditions. Several biological processes are regulated by miRNAs, including cellular differentiation, proliferation, angiogenesis, and apoptosis [19]. In particular, miR-210 is involved in the inhibition of the expression of tumor suppressive genes and in the induction of cell proliferation. In this study, we hypothesize that miR-210 may play a significant role in regulating angiogenesis related to VEGF expression, and VEGF release is conditioned by culturing with or without bone substitute.

Therefore, in the present in vitro study, we isolated and cultured hPDLSCs and examined their morphology, viability, osteogenic differentiation, VEGF release, and miR-210 expression when seeded on Endobon^®^ Xenograft granules (G), a fully deproteinated and sterilized hydroxyapatite ceramic type of scaffold derived from cancellous bovine bone. The ability of hPDLSCs to release VEGF and to express miR-210 when cultivated on the biomaterial was also evaluated. This in an attempt to provide needed information for the potential use of this biocomplex in bone repair and regeneration. The null hypothesis of our study was to obtain no variations in miR-210 expression and VEGF release in hPDLSCs cultured with the biomaterial.

## 2. Results

### 2.1. hPDLSCs Charcterization

**Figure 1:**
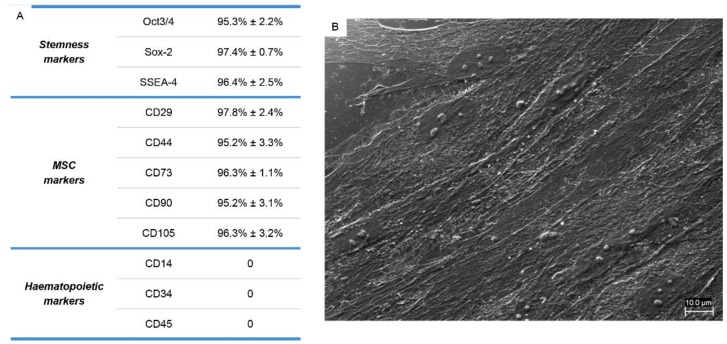
(**A**) Immunophenotypic characterization of human periodontal ligament stem cells (hPDLSCs). Human PDLSCs were positive for typical mesenchymal stem-cell surface markers, but not for hematopoietic markers, including CD14, CD34 and CD45; (**B**) plastic-adherent hPDLSCs observed with Scanning Electron Microscopy (SEM) showed typical fibroblast-like morphological features.

Human PDLSCs, therefore, expressed those MSC surface markers that are usually found in MSCs isolated from other sources. These cells strongly expressed classical MSC markers CD29, CD44, CD73, CD90, and CD105. They also stained positively for pluripotency associated markers OCT3/4, SSEA4, and SOX2. As expected, the cells were negative for the following surface markers: CD14, CD34, and CD45 (Figure 1A). Results are the mean of five different experiments carried out from each different cell culture. Cells seeded in a dish showed normal fibroblast-like morphology, adhered to the bottom dish, and came into contact with the neighboring cells with a long cytoplasmic process, as demonstrated by Scanning Electron Microscopy (SEM) image (Figure 1B).

### 2.2. hPDLSCs and Granule Interaction

**Figure 2:**
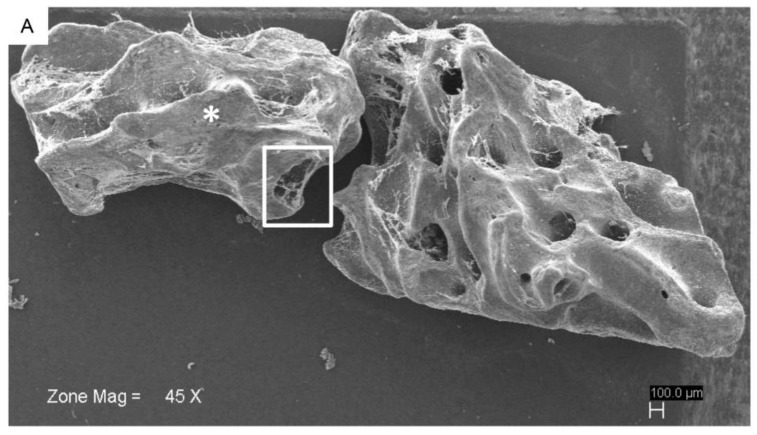

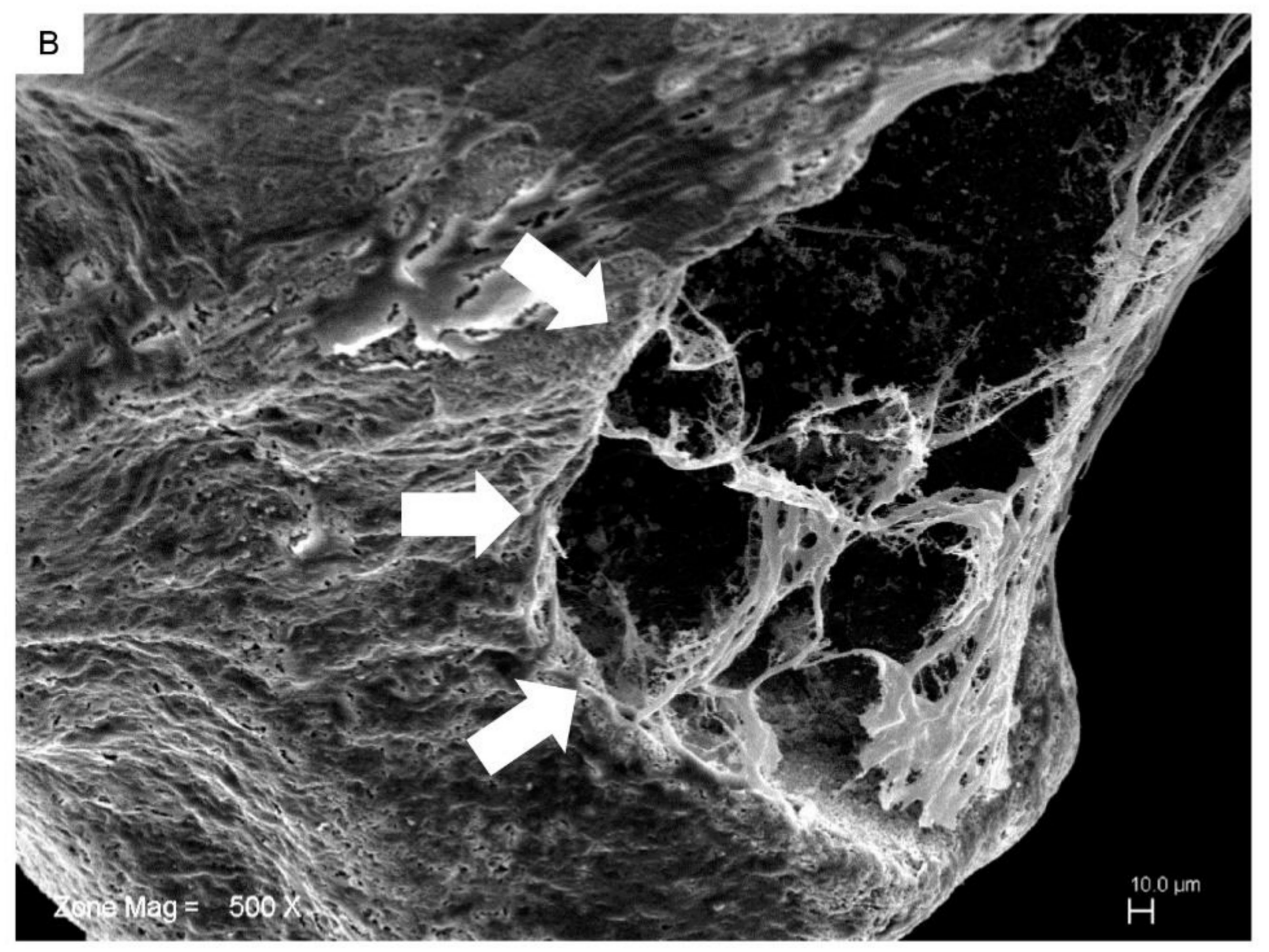
SEM microphotographs of hPDLSCs cultured in the presence of Endobon^®^ Xenograft Granules (G) after 7 days of incubation. (**A**) Human PDLSCs seeded on the G at low magnification (45×); (**B**) SEM image showed hPDLSCs cultured on bone substitute and covered the biomaterial surface with a high interaction between cells and granule surface; in particular, cellular bridges were evident in the cancellous space (arrows) (500×). (* indicated G).

The behavior and morphology of hPDLSCs was examined by SEM and Confocal Laser Scanning Microscopy (CLSM) analysis after 7 days of culture on G. An image obtained at low magnification showed the whole surface of G covered by hPDLSCs (Figure 2A). At high magnification (500×), morphological analysis showed extending cytoplasmic processes and filopodia, which enabled the anchorage of cells (Figure 2B). Cells proliferated on the uneven surface and in the holes of the granules to create bridges across the particles, organizing a multilayer covering the 3D substrate.

**Figure 3:**
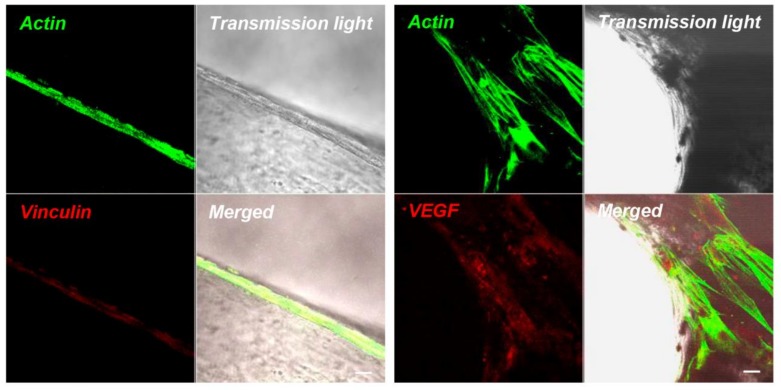
Confocal Laser Scanning Microscopy (CLSM) micrographs of hPDLSCs seeded on granules expressing vinculin molecule. Vinculin (red fluorescence) labeling in cells adhering to the G. Alexa-fluor 488 staining (green fluorescence) showed actin labeling, indicating the spatial cytoskeleton arrangement of hPDLSCs. Granules surface was visible under a light-transmission channel (grey). Merged image of the above-mentioned channel. CLSM micrographs of hPDLSCs seeded on G expressing a positivity for Vascular Endothelial Growth Factor (VEGF). VEGF (red fluorescence) labeling in cells adhering to the G. Alexa-fluor 488 staining (green fluorescence) showed actin-labeling, indicating the spatial cytoskeleton arrangement of hPDLSCs. Granule surface was visible under a light-transmission channel (grey). Merged image of the above-mentioned channel. (* indicated G). Bars: 10 µm.

Immunohistochemistry results displayed the high performance of viable hPDLSCs on G after 7 days of culture. The fluorescent micrograph showed a specific positive response to vinculin, red fluorescent, indicating that an intimate contact between cells and the substrate was established (Figure 3). The actin filaments, green fluorescent, revealed the normal cytoskeleton arrangement. Other than adhesion on bone substitutes, CLSM images showed a positive staining for VEGF of hPDLSCs cultured on granules after 7 days of culture (Figure 3).

### 2.3. Cell Proliferation and Osteogenic Differentiation

**Figure 4:**
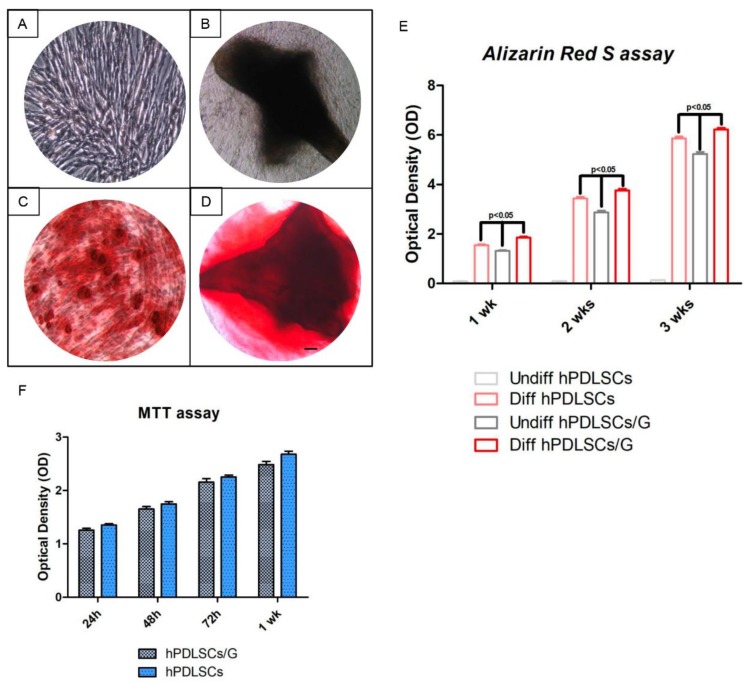
(**A**) HPDLSCs cultured in basal conditions without G and negative for Alizarin Red S (ARS) staining; (**B**) hPDLSCs cultured in basal conditions with G and negative for ARS; (**C**) hPDLSCs cultured in osteogenic conditions without G and positive for ARS; (**D**) hPDLSCs cultured in osteogenic conditions with G and positive for ARS. ARS positive staining was more evident in cells grown under osteogenic conditions and in the presence of granules after 3 weeks of culture; (**E**) graph of ARS staining quantification; (**F**) MTT assay.

The osteogenic differentiation process induced in primary culture with and without a scaffold was evaluated by Alizarin Red S (ARS) staining (Figure 4C,D) and its colorimetric detection (Figure 4E). A progressive increased extracellular deposition was evident in cells grown under an osteogenic condition in comparison with control cells (Figure 4A,B). Differentiation induction were more evident in cells seeded on G cultured with osteoinductive medium, showing the scaffold ability to improve new bone deposition in presence of osteogenic inductors. Moreover, to study the impact of cell proliferation of granules, an MTT assay was performed. Proliferation data of hPDLSCs seeded with G were similar to those of the hPDLSCs cultured without G, used as negative control from 24 h up to 1 week of culture (Figure 4F).

**Figure 5:**
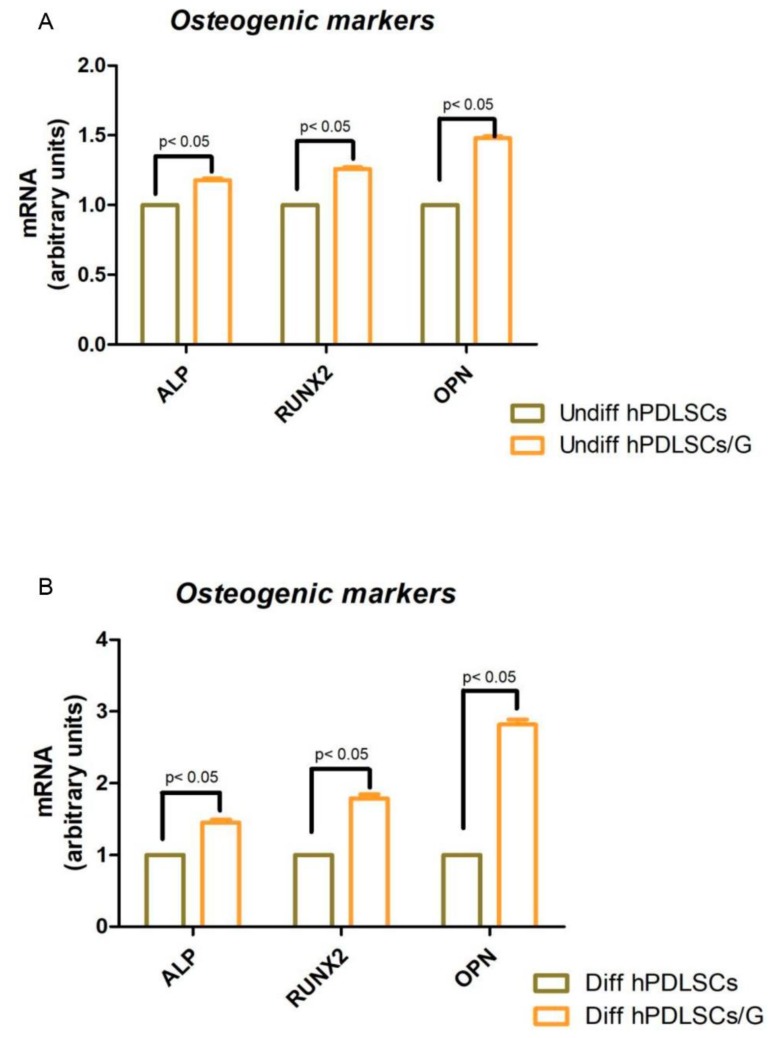
Evaluation of osteogenic markers: ALP, RUNX2 and OPN expression by Real Time PCR assay in undifferentiated (**A**) and differentiated (**B**) hPDLSCs grown in presence or not of G. Data are the mean ± SEM of three separate experiments; *p* < 0.05 was considered statistically significant.

RT-PCR of osteogenesis-related markers showed an upregulation of RUNX-2, ALP and OPN in which were more evident in differentiated hPDLSCs in presence of G when compared with cells cultured without G (Figure 5A). In hPDLSCs cultured in basal conditions (undifferentiated), the osteogenic-related markers were upregulated in cells placed with G (Figure 5B).

**Figure 6:**
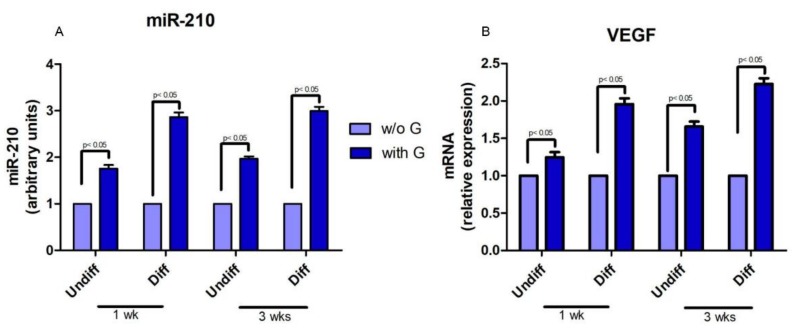
Bar charts show miR-210 (**A**) and VEGF (**B**) expression at 1 and 3 weeks under basal and osteogenic conditions; *p* < 0.05 was considered statistically significant.

miR-210 and VEGF expression was up-regulated in all hPDLSCs grown in presence of granules, both with basal and differentiated medium (Figure 6A,B).

### 2.4. ELISA Test

**Figure 7:**
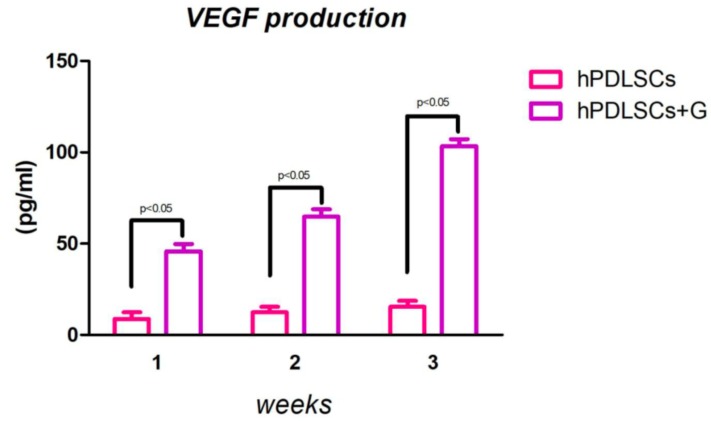
VEGF levels in cellfree-culture supernatants were measured using an ELISA. Each value represents the mean ± SEM of five independent experiments performed in triplicate; *p* < 0.05 was considered different statistically significant from the hPDLSCs seeded with and without G.

VEGF release was detected in culture medium in both experimental conditions. Human PDLSCs were incubated with and without G for 24 h at 37° in a humidified atmosphere at 5% CO_2_. Then, the supernatants were collected to perform an Elisa assay after 1, 2 and 3 weeks of incubation (Figure 7). The results obtained showed an increase of VEGF release when the cells were in presence of G.

## 3. Discussion

Our results showed a logarithmic cell-proliferation rate of hPDLSCs seeded on the biomaterial and the subsequent colonization of the granules’ scaffold observed at SEM and CLSM microscopy; cells contact the uppermost surface, and many cellular bridges between the granules were evident. Moreover, the fluorescent-tagged vinculin, a protein known to crosslink actin filament molecules at focal adhesion [20,21], demonstrated that the focal adhesion area between cells and biomaterial was present. Indeed, numerous anchoring junctions linking hPDLSCs to the 3D granules were evidenced at confocal laser scanning microscopy analysis.

In vitro cell culture offers an ideal instrument to explore specific different biomaterial scaffolds and, in the present study, we successfully constructed novel tissue, engineered using human periodontal ligament stem cells and a granule scaffold.

The basic aspects of bone-tissue engineering, including the chemical composition, roughness, and geometry of the scaffold design, can profoundly affect cell adhesion and maintenance of its proper shape and size. Numerous researchers have demonstrated that the mechanical properties of scaffolds could significantly guide cell migration and stimulate their growth and differentiation [22,23,24,25,26].

To date, stem-cell-based tissue engineering is particularly focused on Bone-Marrow Stem Cells (BMSCs) and Dental Pulp Stem Cells (DPSCs) [27]. We have previously stated that there are no differences between hBMSCs and hPDLSCs in terms of stemness features and multilineage differentiation capacities [28,29,30]. hPDLSCs are easier to obtain than BMSCs, have lower donor-site morbidity, are available in larger numbers, and express stemness markers [31,32]. Thus, we decided to continue this study using periodontal ligament stem cells. In particular, the periodontal ligament contains various types of cells, including PDLSCs and Human Hertwig’s epithelial root sheath/epithelial rests of Malassez (HERS/ERM) cells. The interactions between PDLSCs and HERS/ERM cells could contribute to the homeostasis of the periodontium [33].

Although RT-PCR showed no differences in the gene expression of osteogenic markers, as RUNX-2, ALP and OPN between cells were seeded with and without the scaffold under basal conditions, a significant upregulation of these osteogenic markers was evident when hPDLSCs were cultured on the granules in the presence of osteoinductive conditions. These results indicate that G in basal conditions is not an osteogenic inductor but, when cells are cultured with an inductive osteogenic medium, the ability to differentiate was amplified, suggesting that granules could control a cascade of molecular events involved in bone formation.

MicroRNAs have been widely studied in the regulation of many cellular processes, including proliferation, apoptosis [34], metabolism [35], neuronal patterning [36], and tumorigenesis [37]. miRNAs are also involved in stem-cell functions, such as differentiation, by regulating the post-transcriptional process [38,39]. In our study, we found that miR-210 was upregulated in hPDLSCs grown in the presence of bone substitutes when compared to the cells cultured without granules. Recent evidence indicates that miR-210 play a critical role in cell survival and angiogenesis [40]. Shijun Hu et al. reported that miRNA may have a significant role in regulating angiogenesis and apoptosis after myocardial infarction and could lead to a novel therapy for ischemic heart disease [41]. Our results on miR-210 have been correlated with VEGF mRNA expression and release in the culture medium. RT-PCR and Elisa assay indicated an upregulation of the vascular endothelial growth factor cytokine, measured in the medium after 1, 2 and 3 weeks of culture in the presence of G.

Tissue repair requires the development of a vascular system at the sight of injury for the delivery of oxygen and nutrients; this process, termed “angiogenesis”, is regulated through a complex mechanism of molecular signals mediated by different growth factors. VEGF-A is a key regulator of physiological hemoangiogenesis during development, postnatal growth, and homeostasis. The role of VEGF in the regulation of differentiation pathway of skeletal cells as chondrocytes, osteoblasts, and osteoclasts is not actually well known, even if osteoblast-derived VEGF is critical in bone homeostasis through induction to differentiation of mesenchymal stem cells to osteoblasts [42]. VEGF has a very short half-life in vivo, but its expression is necessary for approximately four weeks for stabilize newly formed vessels [43]. Blood vessels are an important component of bone formation and maintenance, and the rapid vascularization of tissue-engineered osteogenic grafts is a major obstacle in the development of regenerative medicine approaches for bone repair. For this purpose, human bone marrow stem cells have been genetically modified to intensify VEGF expression to generate osteogenic grafts [44]. In effect, VEGF stimulates the chemotactic migration and proliferation of primary human osteoblasts [45], and helps to maintain postnatal bone homeostasis via both intracrine and paracrine functions [42], promoting osteoblast differentiation via intracrine functions, and osteoclast differentiation by the paracrine function [42].

Our results indicate that the 3D granules in contact with hPDLSCs showed not only osteoconductive properties evaluated through adhesion and proliferation, but also the ability to stimulate VEGF secretion from cells related to miR-210 expression. The induction of the production of this growth factor from hPDLSCs could represent a goal for tissue engineering and, in particular, for the therapeutic growth of new blood vessels around the biomaterial in the first phase of osseointegration. Thus, the hPDLSC/G construct could represent an interesting strategy to prefabricate a vascularized bone segment to be transplanted into the defect site.

## 4. Materials and Methods

### 4.1. Scaffold Material

The scaffold material is Endobon^®^ Xenograft (Zimer Biomet, Palm Beach Gardens, FL, USA) granules (G). It is a bovine-derived hydroxyapatite that has been deproteinated by a two-step process. It is also nonresorbable material suited for regeneration in small bone defects. Particle size was in the range of 500–1000 µm [18]. To perform the subsequent experiments, G were placed in multiwell in a ratio of 0.001 g/mm^2^. Cells were seeded in multiwell at a density of 6 × 10^3^/cm^2^; the same cell count was used to be grown on G and incubated at 37 °C in a humidified atmosphere of 5% CO_2_.

### 4.2. Cell Culture

Human periodontal ligament biopsies were collected from 5 different patients after obtaining written informed consent from each participant. The Medical Ethics Committee of the Medical School, G. d’Annunzio University, Italy approved the present study (experimenter: Trubiani Oriana, approval number: n 3/14). All volunteers were exempt from systemic and oral diseases. Biopsies were obtained from alveolar crestal and horizontal fibers of the PDL by scraping with a Gracey’s curette the roots of noncarious third molar teeth. Tissue fragments were placed in a Petri dish with Mesenchymal Stem Cell Basal Medium (MSCBM) (Lonza Walkersville Inc., Walkersville, MD, USA) and maintained at 37 °C in a humidified atmosphere at 5% CO_2_. After 1 week of incubation, hPDLSCs were spontaneously migrated from the tissue and cultured in MSCBM (Lonza, Basel, Switzerland) as previously described by Diomede et al. [46]. Plastic-adherent cells at 80%–90% confluent were observed by phase-contrast microscopy, then isolated using 0.1% trypsin solution, and plated in tissue culture polystyrene flasks at 5 × 10^3^ cells/cm^2^. Primary cultures of hPDLSCs were constituted of colonies of fibroblast-like cells that, after subcultivation, proliferated with a population-doubling time of 48 h, reaching a confluent growth-arrested condition. Single-cell suspensions of the developing adherent layer were prepared by trypsin/ethylenediaminetetraacetic acid (EDTA) (Lonza). The following experiments were repeated for every hPDLSCs culture derived from each patient.

### 4.3. Flow-Cytometry Analysis

HPDLSCs were washed in PBS and subsequently resuspended in PBS with saturating concentrations (1:100 dilution) of fluorescein isothiocyanate-conjugated antihuman antibodies (CD44, CD45, CD73, and CD90) and phycoerythrin-conjugated CD14, CD29, CD34, and CD105 for 30 min at 4 °C. Labelled cells were acquired and analyzed using an FACStar-plus flow-cytometry system running CellQuest software (Becton-Dickinson, Mountain View, CA, USA). All reagents were obtained from Becton Dickinson.

### 4.4. MTT Assay

The proliferation rate of hPDLSCs seeded with or without G was measured by quantitative colorimetric MTT (3-[4,5-dimethyl-2-thiazolyl]-2,5-diphenyl-2*H*-tetrazoliumbromide test) (Promega, Milan, Italy) as previously reported [47]. Briefly, 2.5 × 10^5^ cells/well were seeded into a 96-well culture plate with MSCBM medium (Lonza), after 24 h till 1 week of incubation at 37 °C, 15 μL/well of MTT was added to culture medium, and cells were incubated for 3 h at 37 °C. The supernatants were read at 650 nm wavelength using an ND-1000 NanoDrop Spectrophotometer (NanoDrop Technologies, Rockland, DE, USA). Cells cultured without G were used as negative control. The MTT assay was performed in three independent experiments, 6 replicate wells for each experimental point.

### 4.5. SEM Analysis

Human PDLSCs at second passage and hPDLSCs seeded on G were fixed for 4 h at 4 °C in 4% glutaraldehyde in 0.05 M phosphate buffer (pH 7.4), dehydrated in increasing ethanol concentrations, and then critical-point-dried. They were then mounted on aluminum stubs and gold-coated in an Emitech K550 (Emitech Ltd., Ashford, UK) sputter-coater [48]. Samples were observed with SEM to elucidated the interaction cells-biomaterial (ZEISS EVO 50, Jena, Germany).

### 4.6. Immunofluorescence Staining and CLSM Analysis

To better understand and evaluate the adhesion on G, the hPDLSCs on granules were processed for immunofluorescence labeling. Samples were fixed for 10 min at room temperature (RT) with 4% paraformaldehyde in 0.1M sodium phosphate buffer (PBS), pH 7.4. Then, samples were permeabilized with 0.5% Triton X-100 in PBS, followed by blocking with 5% skimmed milk in PBS. Primary monoclonal antibodies to antivinculin (1:500; Santa Cruz Biotechnology, Santa Cruz, CA, USA) and anti-VEGF (1:250; Santa Cruz Biotechnology) were used, followed by Alexa Fluor 568 green fluorescence conjugated goat antimouse as secondary antibodies (Molecular Probes, Invitrogen, Eugene, OR, USA). Subsequently, samples were incubated with Alexa Fluor 488 phalloidin green-fluorescence conjugate (Molecular Probe) as cytoskeleton actin marker. Samples were placed face-down on glass slides and mounted with Prolong antifade (Molecular Probes) [49]. Samples were observed with CLSM (Zeiss LSM510META) and connected to an inverted Zeiss Axiovert 200 microscope equipped with a Plan Neofluar oil-immersion objective (40×/1.3 NA).

### 4.7. Osteogenic Differentiation

For osteogenesis induction, the primary cells at second passage were seeded at 4 × 103 cells /cm^2^ in an MSCBM culture medium and maintained in the culture at 37 °C, in a humidified atmosphere of 5% CO_2_. After reaching 80% confluence, cells were incubated with MSCBM medium with the addition of osteogenic supplements, i.e., 100 nM dexamethasone (Applichem GmbH, Darmstadt, Germany), 10 nM β-glycerol-phosphate (Applichem), and 0.05 mM 2-phosphate-ascorbic acid (Sigma-Aldrich, Milan, Italy) [50]. Confluent cells with and without G were induced to osteogenic differentiation. Visualization of calcium deposition and extracellular matrix mineralization was obtained by ARS staining assay performed after 1, 2 and 3 weeks of culture. This test was carried out according to the method described by Libro et al. [51]. Cells were washed with PBS, fixed in 10% (*v*/*v*) formaldehyde (Sigma-Aldrich) for 30 min, and washed twice with abundant dH2O prior to the addition of 0.5% Alizarin red S in H_2_O, pH 4.0, for 1 h at room temperature.

After cell incubation under gentle shaking, cells were washed with dH2O 4 times for 5 min. For staining quantification, 800 μL 10% (*v*/*v*) acetic acid was added to each well. Cells were incubated for 30 min with shaking, and then scraped from the plate, transferred into a 1.5 mL vial, and vortexed for 30 s. The obtained suspension, overlaid with 500 μL mineral oil (Sigma-Aldrich), was heated to 85 °C for 10 min, then transferred to ice for 5 min, carefully avoiding opening the tubes until fully cooled, and centrifuged at 20,000× *g* for 15 min [52]. Five hundred microliters of the supernatant was placed into a new 1.5 mL vial, and 200 μL of 10% (*v*/*v*) ammonium hydroxide was added to neutralize the acid, assuring a pH between 4.1 and 4.5. One-hundred and fifty microliters of the supernatant obtained from differentiated and undifferentiated hPDLSCs grown with or without G was read in triplicate at 405 nm by a spectrophotometer (Synergy HT). The osteogenic induction was performed in three independent experiments for each experimental group, and spectrophotometer reads were normalized per cell number.

### 4.8. RNA Isolation and Real-Time PCR Analysis

Osteogenic markers were evaluated by real-time PCR. To this end, total RNA was isolated using the Total RNA Purification Kit (Norgen Biotek Corp., Ontario, CA, USA) according to the manufacturer’s instructions. M-MLV Reverse Transcriptase reagents (Applied Biosystems) were used to generate cDNA. Six micrograms RNA for each sample was used. Real-time PCR was carried out with the Mastercycler ep realplex real-time PCR system (Eppendorf, Hamburg, Germany). HPDLSCs expression of runt-related transcription factor-2 (RUNX-2), alkalin phospatase (ALP), osteopontin (OPN), and VEGF were evaluated. Expression levels in cells cultured with the basal medium and with differentiating medium in the presence or absence of G were compared. Commercially available TaqMan Gene-Expression Assays (RUNX-2 Hs00231692_m1; ALP Hs01029144_m1; OPN Hs00157093_m1) and the TaqMan Universal PCR Master Mix (Applied Biosystems, Foster City, CA, USA) were used according to standard protocols. Beta-2 microglobulin (B2M Hs99999907_m1) (Applied Biosystems, Foster City, CA, USA) was used for template normalization. RT-PCR was performed in three independent experiments, and duplicate determinations were carried out for each sample [53].

### 4.9. MicroRNAs Quantization

miRNA were extracted after 1 and 3 weeks of culture using the PureLink RNA mini kit (Life Tech), treated with the RNase-Free DNase Set (Qiagen, Venlo, The Netherland) according to the instructions of the manufacturer and quantified with Nanodrop2000 (Thermo-Scientific, Waltham, MA, USA). Gene sequences were from NCBI (http://www.ncbi.nlm.nih.gov), and RNA sequences for miR-21 were used into the Universal ProbeLibrary (UPL) Assay Design Center software (https://www.rocheappliedscience.com) to identify primers and UPL probe. Total RNA (50–200 ng) was retrotranscribed with High-Capacity cDNA Reverse-Transcription Kit (Life Technologies, Milan, Italy). MicroRNA quantization was performed using stem-loop RT primers designed with a modification to include the UPL #21 sequence-binding site [54]. UPL probe #21 was from the UPL database (Roche Diagnostics, Basel, Switzerland). Total RNA (50 ng) was retrotranscribed with a TaqMan MicroRNA Reverse-Transcription Kit (Life Technologies). Reactions were incubated for 30 min at 16 1 °C, followed by pulsed RT of 60 cycles at 30 1C for 30 s, 42 1C for 30 s, and 50 1C for 1 s. Real-time PCRs were performed in an Applied Biosystems 7900 instrument. miRNA and mRNA levels were measured using Ct (threshold cycle). The target amount, normalized to endogenous reference 18S/RNU44 and relative to a calibrator, was given by 2 DDCt and/or 2 DCt methods (Life Technologies).

### 4.10. ELISA Test

A number of HPDLSCs, 2 × 10^4^, cultured in MSCBM medium without FBS (Lonza Verviers Company, Verviers, Belgium), were seeded with and without G, and incubated at 37 °C in humidified air at 5% CO_2_. Supernatants of cell culture maintained in the presence or in the absence of G were collected after 1, 2, and 3 weeks of culture and, subsequently, VEGF expression (R and D System, Minneapolis, MN, USA) by ELISA assay was performed. The supernatants were normalized according to the cell number [55]. The ELISA assay was performed in three independent experiments, with three replicate wells for each experimental point.

### 4.11. Data and Statistical Analysis

The Statistical Package for Social Science (SPSS, v.21.0, Inc. Chicago, IL, USA) was used for data analysis. Parametrical methods were used after verifying the existence of the required assumptions. In particular, the normality of the distribution and the equality of variances were assessed by the Shapiro-Wilk and Levene’s tests, respectively. The factors under investigation were the time elapsed and the presence of granules for the MTT assay, osteogenic differentiation, and VEGF release. Data were expressed as means and standard deviation of the recorded optical density values. The differences among the levels of the two factors under investigation were evaluated performing three distinct two-way ANOVA tests, one for each experiment. Tukey tests were applied for pairwise comparisons. A value of *p* < 0.05 was considered statistically significant in all tests.

## 5. Conclusions

Our results indicate that the 3D granules, in contact with hPDLSCs, showed not only osteoconductive properties, evaluated through the adhesion and proliferation process, but also the ability to stimulate VEGF secretion in hPDLSCs via miR-210 involvement. The induction of the production of this growth factor from hPDLSCs could represent a goal for tissue engineering, in particular for the therapeutic growth of new blood vessels around the biomaterial in the first phase of osteointegration. Thus, the hPDLSCs/G construct could represent an interesting strategy to prefabricate a vascularized bone segment to be transplanted into the defect site. Moreover, the identification of miR-210 involved in the VEGF secretion pathway could offer a novel regulatory system in the early steps of the bone-regeneration process.

## Abbreviations

G	Granules
MSCs	Mesenchymal Stem Cells
hPDLSCs	Human Periodontal Ligament Stem Cells
SEM	Scanning Electron Microscopy
hGMSCs	Human Gingival Mesenchymal Stem Cells
VEGF	Vascular Endothelial Growth Factor
miRNA	microRNA
BMSCs	Bone-Marrow Stem Cells
3D	Three-dimensional
ALP	Alkalin Phospatase
OPN	Osteopontin
RUNX-2	Runt-related Transcription Factor-2
ARS	Alizarin Red S
EDTA	Eethylenediaminetetraacetic acid
